# Content and Functionality of Alcohol and Other Drug Websites: Results of an Online Survey

**DOI:** 10.2196/jmir.1449

**Published:** 2010-12-19

**Authors:** Britt Klein, Angela White, David Kavanagh, Kerrie Shandley, Frances Kay-Lambkin, Judith Proudfoot, Judy Drennan, Jason Connor, Amanda Baker, Ross Young

**Affiliations:** ^7^School of Advertising, Marketing and Public RelationsFaculty of BusinessQueensland University of TechnologyBrisbaneAustralia; ^6^BlackDog Institute & School of PsychiatryUniversity of New South WalesSydneyAustralia; ^5^Centre for Brain and Mental Health ResearchFaculty of HealthUniversity of NewcastleNewcastleAustralia; ^4^National Drug and Alcohol Research CentreUniversity of New South WalesSydneyAustralia; ^3^Institute of Health and Biomedical InnovationSchool of Psychology and CounsellingQueensland University of TechnologyBrisbaneAustralia; ^2^Centre for Youth Substance Abuse ResearchFaculty of Health SciencesUniversity of QueenslandBrisbaneAustralia; ^1^National eTherapy CentreFaculty of Life and Social SciencesSwinburne UniversityMelbourneAustralia

**Keywords:** Alcohol, drugs, Internet, online survey, stress, health, website interactivity, website trustworthiness, Web-based interventions

## Abstract

**Background:**

There is a growing trend for individuals to seek health information from online sources. Alcohol and other drug (AOD) use is a significant health problem worldwide, but access and use of AOD websites is poorly understood.

**Objective:**

To investigate content and functionality preferences for AOD and other health websites.

**Methods:**

An anonymous online survey examined general Internet and AOD-specific usage and search behaviors, valued features of AOD and health-related websites (general and interactive website features), indicators of website trustworthiness, valued AOD website tools or functions, and treatment modality preferences.

**Results:**

Surveys were obtained from 1214 drug (*n* = 766) and alcohol website users (*n* = 448) (mean age 26.2 years, range 16-70). There were no significant differences between alcohol and drug groups on demographic variables, Internet usage, indicators of website trustworthiness, or on preferences for AOD website functionality. A robust website design/navigation, open access, and validated content provision were highly valued by both groups. While attractiveness and pictures or graphics were also valued, high-cost features (videos, animations, games) were minority preferences. Almost half of respondents in both groups were unable to readily access the information they sought. Alcohol website users placed greater importance on several AOD website tools and functions than did those accessing other drug websites: online screening tools (χ²_2_ = 15.8, *P* < .001, n = 985); prevention programs (χ²_2_ = 27.5, *P* < .001, n = 981); tracking functions (χ²_2_ = 11.5, *P* = .003, n = 983); self help treatment programs (χ²_2_ = 8.3, *P* = .02, n = 984); downloadable fact sheets for friends (χ²_2_ = 11.6, *P* = .003, n = 981); or family (χ²_2_ = 12.7, *P* = .002, n = 983). The most preferred online treatment option for both the user groups was an Internet site with email therapist support. Explorations of demographic differences were also performed. While gender did not affect survey responses, younger respondents were more likely to value interactive and social networking features, whereas downloading of credible information was most highly valued by older respondents.

**Conclusions:**

Significant deficiencies in the provision of accessible information on AOD websites were identified, an important problem since information seeking was the most common reason for accessing these websites, and, therefore, may be a key avenue for engaging website users in behaviour change. The few differences between AOD website users suggested that both types of websites may have similar features, although alcohol website users may more readily be engaged in screening, prevention and self-help programs, tracking change, and may value fact sheets more highly. While the sociodemographic differences require replication and clarification, these differences support the notion that the design and features of AOD websites should target specific audiences to have maximal impact.

## Introduction

It is estimated that over a quarter of the world’s population use the Internet [1] and that 75% of Internet users have searched for health or medical information on the Web [2]. While the health sector has primarily employed the Internet as a psycho-educational portal, advances in interactive technology have increased the potential of the medium to be used to deliver targeted health interventions and other behavior change programs [3].

While there is a growing trend to use the Internet to deliver alcohol and other drug (AOD) information and resources, little is known as to how best engage “at-risk” populations, such as young people, or how to optimize its access and utilization. Given the appeal of the Internet to young people [4,5] and that this is a group that frequently engages in problematic drinking and does not typically access standard AOD services, targeted programs on the Internet may be a medium that could be employed to great effect in this area.

Much of the published literature concerning online AOD interventions is descriptive [6], providing only general information on program evolution, application, and usage [eg, 5,7-11]. Overall, the findings have underscored the scope and access this medium can offer relating to AOD information, as well as the potential of the Internet for the dissemination of screening, assessment, and intervention programs. Some studies [eg, 10,11] have found that individuals unconstrained by geographic location, access Internet-based AOD information and resources in numbers that would overwhelm a traditional face-to-face health service. For example, a naturalistic Internet-based tobacco cessation study by Saul et al [9] reported that in 2 months 100,000 people visited the program website and over 23,000 registered with the program. A study by Linke et al [12] reported an average of 1039 visits per month to the “Down Your Drink” website drawn from over 41 countries. Furthermore, Internet-available information and services appeal to diverse populations including women and young people, who do not necessarily access standard face-to-face AOD services. For example, Koski-Jännes et al [13] found that 61% of those who accessed the “Drinking Habit Test” were women.

In relation to the reasons for use of online AOD resources and materials, out-of-hours availability has been found to be important [5,14]. Other reasons for use of online AOD resources include ease of access to a computer, the anonymity and privacy afforded by the medium, and not having to attend face-to-face meetings [14]. However, Internet programs that require repeated access or extended periods of engagement on the site experience high dropout rates [5,15].

Research into how people search and engage with health websites suggests that the typical user explores only the first few links on a search engine and assesses website credibility by the source of the information cited on the Web page and the professionalism of the website program design [16]. Eysenbach and Köhler [16] noted that in observational studies, Internet users rarely checked the "about us" sections of websites, investigated who the authors or owners of the site were, or read disclaimers or disclosure statements. Perhaps even more telling was the finding that very few Internet users remembered which websites they had retrieved information from or who had developed the sites [16].

The way in which the information is presented can mediate the duration and frequency of visits to a website. For example, the website itself, along with the navigation configuration, needs to be attractive and easy to use [17,18]. Less structured websites do not hold visitors, and websites that do not change over time attract fewer repeat visits [19]. Individuals have been found to be more likely to stay longer at sites that provided personalized feedback as well as relevant and reliable information [18].

While general Internet health access and usage is an important starting point for AOD website design, currently there is a lack of information on users’ knowledge, experience, and opinions of AOD websites. There is a need to gain an understanding of site users’ preferences and perceived gaps or deficiencies of existing sites if this medium is to be of optimal value as an AOD health promotion, prevention, and intervention tool. The aim of the current study was to address this gap in the existing research and investigate the experiences of AOD website users and their views about the content, functionality, and utility of these websites.

## Methods

An open-access online survey was developed to examine Internet use and opinions of AOD and health-related websites. The current paper focuses on results relating to AOD websites only.

### Development of Survey Themes and Specific Questions and Pretesting

Initial development of the survey involved face-to-face, teleconference, and email discussion between members of the research team in order to formulate the themes and specific questions. The draft survey was submitted to the project advisory group, consisting of government and nongovernment AOD, youth, and primary care representatives, for review and comment. The final survey explored the following themes:

1. General Internet usage
2. AOD website Internet usage and search behaviours
3. Most-visited AOD websites
4. General website features
5. Interactive website features
6. Judging website trustworthiness
7. Preferences regarding AOD websites tools and functions
8. Preferences for AOD online treatment modalities and support

The survey was pilot tested online (via Survey Monkey) by members of the research team and several independent community members. Usability and functionality were improved prior to online administration.

### Online Survey Design and Administration

Adaptive questioning features were employed to avoid survey respondents having to answer unnecessary questions. The presented questions, therefore, depended on answers to prior questions (eg, if respondents had never visited an AOD website, they were not asked further questions about theses websites). There were no more than four questions to a Web page (averaging two questions per page), spanning a potential 84 Web pages. Although there were 188 questions in the total survey, the adaptive questioning procedures meant that the maximum number of questions presented to a respondent was 118. All questions were forced choice, that is, to proceed to the next Web page, all responses to all questions on the preceding page were required. As there was no “back” button offered, respondents could not change answers on previous Web pages. Survey Monkey captured question responses automatically and placed them into an electronic database (Excel) that could only be accessed by the account creator.

### Consent Process and Advertising

The survey was voluntary, anonymous, and took 10 to 15 minutes to complete. Upon entry to the survey home Web page, respondents were provided with an explanatory statement outlining the study (eg, purpose of the study, funding source, name of the chief investigator, length of survey, data storage, and ethics committee approval) and were asked to provide online informed consent prior to accessing the survey questions. Respondents were also offered the chance to enter a draw to win an 8GB iPod Nano for completing the survey if they provided their name and an email address. Respondents were informed that identifying information would be stored separately from their survey data. Survey data was collected in a one-month time frame (March-April 2009). Ethics approval was obtained from the Queensland University of Technology Human Ethics Research Committee.

The survey and Web link was advertised via Facebook, AOD and health-related websites, and a range of industry, consumer, and tertiary institutional email lists. A copy of the survey announcement can be found in Multimedia Appendix 1.

## Results

### Response Rate

To be eligible to participate, respondents were required to be an Australian resident and at least 16 years old. (Ages of respondents ranged from 16 to 70 years.) Of the 3313 people who accessed the survey, 305 were excluded for the following reasons: discontinuation at the information statement (n *=* 11); failure to give consent (n *=* 124); not responding to any survey questions (n *=* 167); resided overseas (n *=* 2); and being under 16 years of age (n *=* 1). Of 3008 respondents, 1794 had visited health-related websites but had not previously visited an AOD website. These respondents were therefore not included in the analyses reported in this paper. The remaining 1214 respondents had endorsed visiting either an alcohol website (448/1214, 36.9%) or a drug website (766/1214, 63.1%), were unique visitors (based on their IP addresses), and had responded to at least one survey question. The majority completed the entire survey (882/1214, 72.7%).

### Demographic Information

Table 1 provides demographic data for the drug and alcohol website user groups.

**Table 1:** Percentages and n values for gender, education, relationship status, employment status, English as a first language, and age of drug website users and alcohol website users

Demographic Variable		Type of Website Accessed				
		Drug Website Users			Alcohol Website Users			
		%	n	%	n	%	N
**Gender**							
	Male	35	193	32.4	108	34	301
	Female	65	359	67.6	225	66	584
**Completed secondary education**		91.5	505	92.2	307	91.8	812
**Level of tertiary education**							
	None	7.8	43	6.3	21	7.2	64
	Apprenticeship/trade	1.6	9	1.5	5	1.6	14
	Other certificate	3.1	17	3.9	13	3.4	30
	Diploma	5.3	29	3.6	12	4.6	41
	Current undergraduate	57.8	319	59.6	198	58.5	517
	Completed undergraduate	12.7	70	11.7	39	12.3	109
	Current postgraduate	1.3	7	0.9	3	1.1	10
	Completed postgraduate	10.1	56	12.3	41	11.0	97
	Other	0.4	2	0	0	0.2	2
**Relationship status**							
	Single	46.4	225	49.1	163	47.4	418
	Married/cohabitating with partner	28.4	156	26.2	87	27.6	243
	In relationship, not cohabitating	23.3	128	22.9	76	23.1	204
	Divorced/separated and single	1.8	10	1.8	6	1.8	16
	Widowed and single	0.2	1	0	0	0.1	1
**Employment status**							
	Employed full-time	18.3	101	20.8	69	19.3	170
	Employed part-time/casual	53	292	52.1	173	52.7	465
	Home duties	2.4	13	2.4	8	2.4	21
	Disability support	0.4	2	0.6	2	0.5	4
	Unemployed	12	66	10.2	34	11.3	100
	Retired	0.2	1	0.6	2	0.3	3
	Student	10.5	58	10.2	34	10.4	92
	Student and working	2.2	12	2.1	7	2.2	19
	Self-employed	0.7	4	0.6	2	0.7	6
	Other	0.4	2	0.3	1	0.3	3
**English as a first language**		85.3	469	88.3	293	86.4	762
**Age category**							
	16-24	65.9	405	63.3	228	64.9	633
	25-33	17.6	108	18.1	65	17.7	173
	34-43	6.5	40	8.3	30	7.2	70
	44-52	7.3	45	6.1	22	6.9	67
	53-61	1.6	10	3.1	11	2.2	21
	62-70	1.1	7	1.1	4	1.1	11

There were no significant differences between the alcohol website users and the drug website users on any demographic variable: gender (Fisher exact test χ²_1_ = 0.6, *P* = .46, n = 885); education (χ²_9_ = 4.9, *P* = .80, n = 884); relationship status (χ²_4_ = 1.3, *P* = .86, n = 882); employment status (χ²_9_ = 2.7, *P* = .98, n = 883); English as a first language (Fisher exact test χ²_1_ = 1.6, *P* = .23, n = 882); age category (χ²_5_ = 3.95, *P* = .56, n = 975). There was also no difference in the mean ages in years of the users of the alcohol website (26.3, SD 10.4) and the users of the drug website (26.0, SD 9.9) (t_1,973_ = .205, *P* = .65). The median age of both the alcohol and drug groups was 22.0 years.

### General Internet Usage

Drug and alcohol website users reported primarily accessing the Internet from home (959/1214, 79.4%) or at university, school, or work (232/1214, 18.7%), via cable broadband (306/1214, 39.9%), ADSL (243/1214, 20.0%), or ADSL2 (185/1214, 24.2%). Respondents commonly accessed the Internet daily (1178/1214, 97.0%) and were typically online for periods ranging from 5 to 30 minutes (217/1214, 17.9%), 30 to 60 minutes (316/1214, 26.0%), 1 to 2 hours (315/1214, 25.9%), or 2 or more hours (366/1214, 30.1%). Daily online activities were email (1060/1209, 87.7%), social networking, (eg, Facebook and MySpace) (688/1203, 57.2%), news (574/1197, 48%), and random “surfing” (506/1200, 42.2%). Over 90% (1112/1214, 91.6%) said they felt comfortable/confident when using the Internet. There were no significant differences between the alcohol and drug website user groups on any of these general Internet usage variables.

### Alcohol and Other Drug Websites Usage and Search Behaviours

Most respondents found the websites via search engines (610/766 or 79.6% of drug website users and 341/448 or 76.1% of alcohol website users). Both groups were primarily interested in finding information about effects of the substance used (688/740 or 93.0% of drug group and 307/421 or 72.9% of alcohol group).

When respondents chose to specify the information they looked for on websites, 74 drug website users reported searching for information on the chemical composition of drugs, firsthand drug user accounts, why people used drugs, health risks and side effects of using drugs, harm minimization strategies, referral links to supports, online assessment, self-help programs, general usage statistics, or drug sentencing laws. The responses of 60 alcohol website users stated that they were looking for information about standard drinks, alcohol content in cocktails and safe drinking limits, alcohol use in pregnancy, alcohol and violence, effects of combining drugs and alcohol, short and long term health issues associated with drinking, hangover cures, relapse prevention information, or reasons why people drink.

Finding the desired information appeared to be difficult for respondents searching alcohol websites. Rating the success of their search on a 3-point scale (yes, somewhat, no), almost half (211/431, 49%) reported they were only somewhat successful in finding what they wanted, and only 47.3% (204/431) said they did find it. Percentages for respondents searching drug-related websites were similar (348/745, 46.7% somewhat; 392/745, 52.6% yes). Just over half of respondents in the drug group (408/736, 55.4%) and alcohol group (232/414, 56.0%) reported being able to source information they wanted within 5 to 15 minutes.

### Most Visited Alcohol and Other Drug Websites

AOD website users were presented with 14 AOD websites (see Multimedia Appendix 2 for AOD website list). They were asked if they had visited each site, and if so, were asked to rate how easy it was to use, its attractiveness, and trustworthiness as very, somewhat, or not at all. The two most visited AOD websites across groups were the Australian Government National Drugs Campaign website (170/727 or 23.4% of the drug group and 110/421 or 26.1% of the alcohol group visited this site) and the National Drug and Alcohol Research Centre website (232/736 or 32.0% of the drug group and 95/421 or 22.6% of the alcohol group visited this site). Interestingly, although both groups rated these websites as very or somewhat easy to use and trustworthy, the ratings regarding the attractiveness of these websites were far lower.

### General Website Features

The drug and alcohol website user groups were presented with a series of general website features and asked to rate how important (very, somewhat, or not at all) they thought these features were for AOD and health-related websites. As shown in Table 2, seven of the listed features were considered very important by the majority of respondents from both groups, with a glossary and sitemap receiving the lowest importance rating. There were no significant differences between the groups on any of the general website features.

**Table 2:** General website features rated very important

General Website Features Rated Very Important	Drug Website Users		Alcohol Website Users	
	% (n)	N	% (n)	N
Easy navigation	88.5 (676)	764	88.3 (392)	444
Open access	87.1 (666)	765	86.2 (381)	442
The right amount of information	82.1 (624)	760	81.2 (358)	441
Internal search function	79.4 (608)	766	79.1 (351)	444
Easy to understand language	75.7 (575)	760	77.9 (346)	444
No need for extra software	73.9 (564)	763	73.5 (324)	441
Interesting Web pages	50.7 (386)	762	56.0 (248)	443
Does not require a high bandwidth	46.3 (353)	761	47.3 (209)	442
Attractive website layout	40.1 (307)	765	45.9 (204)	444
A glossary	30.6 (232)	758	34.2 (151)	442
A sitemap	27.9 (212)	761	26.4 (117)	443

There were no significant differences in the preferences of men and women for general website features. However, the relative importance of a site map (χ²_10_ = 19.8, *P* = .03, n = 969) varied according to age group categories. This result indicated that those aged 44 to 61 years valued site maps significantly more than the other groups. Respondents holding an “other certificate” as their highest education qualification valued easy navigation the least of the education categories (χ²_16_ = 27.5, *P* = .04, n = 879).

### Interactive Website Features

The drug and alcohol website user groups were presented with a series of interactive website features and asked to rate how important (very, somewhat, or not at all) they thought these features were for AOD and health-related websites. As Table 3 shows, being able to print or download information, being able to ask a question, external links, and pictures and graphics were valued most highly, followed by automated personal feedback, video, quizzes, and flash animations. Games, blogs, SMS, or email reminders were less common preferences. There were no significant differences between the two groups on any interactive website feature.

**Table 3:** Interactive website features rated very important

Interactive Website Features Rated Very Important	Drug Website Users		Alcohol Website Users	
	% (n)	N	% (n)	N
Print/download information	68.8 (526)	765	72.5 (321)	443
Being able to ask a question	57.3 (437)	763	58.2 (258)	443
External links	52.5 (400)	762	54.9 (242)	441
Pictures and graphics	46.8 (357)	763	51.8 (230)	444
Automated personal feedback	23.6 (179)	759	26.9 (119)	442
Video	16.1 (123)	762	17.0 (75)	442
Quizzes	15.3 (117)	763	19.7 (87)	442
Flash / Animations	11.3 (86)	760	10.6 (47)	442
Audio	11.3 (86)	763	10.9 (48)	442
Access to a chat room	11.1 (85)	763	12.7 (56)	441
SMS or email reminders	7.7 (58)	757	9.6 (42)	437
Blogging	8.7 (66)	759	6.8 (30)	439
Games	4.5 (34)	758	7.0 (31)	441

Of the all respondents, 31 specified other important general and interactive website features not listed in Table 2. The most common responses were being able to post comments, access to a chat room with an expert to answer questions, online forums to ask anonymous questions, a “frequently asked questions” section, peer testimonials, real facts, polls and votes, and a “where to get help” section.

Preferences for specific interactive website features did not differ according to gender or educational level. However, being able to ask a question was influenced by age group (χ²_10_ = 36.1, *P* < .001, n = 968). The youngest age group valued being able to ask a question most highly, and those between 44 and 61 years of age valued this feature the least. The youngest age group also significantly valued chat room access the most, and those between 44 and 52 years of age valued it least (χ²_10_ = 38.9, *P* < .001, n = 968). Similarly, the option to blog was most important to respondents between 16 and 24 years of age and least important for those aged 25 to 33 (χ²_10_ = 53.3, *P* < .001, n = 962). Furthermore, respondents 16 to 24 years of age valued games the most, and those aged 25 to 33 years old valued them least (χ²_10_ = 27.8, *P* = .002, n = 965). In contrast, the youngest age category valued downloading of information least, and those between 33 and 43 and those between 53 and 61 years of age valued it most (χ²_10_ = 18.6, *P* = .046, n = 968).

### Judging Website Trustworthiness

Respondents were asked to rate the importance (very, somewhat, or not at all) of a number of trustworthiness indicators when they judged whether or not they could trust a website. The percentage of trustworthiness indicators judged as very important are provided in Table 4.

**Table 4:** Indicators of website trustworthiness judged very important

Indicators of Website Trustworthiness Judged Very Important	Drug Website Users		Alcohol Website Users	
	% (n)	N	% (n)	N
It provides evidence for its claims	86.8 (664)	765	87.3 (385)	441
It says where it got its information from	81.3 (621)	764	81.9 (363)	443
There is enough information to tell whether the writers are experts	71.7 (548)	764	75.5 (335)	444
It tells you when it was created or last updated	70.4 (539)	766	72.7 (322)	443
I can easily find who owns and wrote the website	67.9 (518)	763	73.3 (324)	442
It tells you it has a privacy policy	55.5 (422)	761	58.1 (257)	442
It tells you whether sponsors are involved	52.7 (400)	759	53.2 (236)	444
Past experience	50.4 (384)	762	51.8 (228)	440
Has reference or links to other websites	46.4 (354)	763	50.3 (222)	441
It displays a quality seal of approval (eg, HONcode)	44.3 (337)	761	47.1 (209)	444
It has been recommended to me by my peers	37.9 (289)	762	42.1 (187)	444
It has been recommended to me by my family	32.8 (249)	760	37.2 (165)	443
Another site said it was good	18.2 (138)	757	20.1 (89)	442

Over 80% of respondents identified the provision of evidence for claims made on a website and statements describing the source of information provided as very important factors for judging website trustworthiness. No significant differences were found between the two groups on any indicator of trustworthiness.

In addition, 25 respondents were able to specify other important indicators of trustworthy websites not listed in Table 4. The most common responses were: the website was recommended to them by pharmacists, doctors, or counsellors; the website was government affiliated; the contact details of the website owner were provided; the website had a certain ”look and feel” and a “serious tone” and the content was objective and unbiased.

### Opinions About Preferred Website Tools and Functions When Using Alcohol and Other Drug Websites

Alcohol and drug website user groups were asked to consider whether they would use a range of website tools/functions and how important (very important, somewhat, or not at all) these features would be. The percentages of respondents endorsing the tool/function as very important are presented in Table 5.

**Table 5:** Alcohol and drug website tools/functions rated very important

Website Tools/Functions	Drug Website Users		Alcohol Website Users	
	% (n)	N	% (n)	N
Downloadable fact sheets for consumers	55.6 (348)	626	62.2 (224)	360
A Web portal site that has information on the best site and treatment options	49.5 (308)	622	51.5 (185)	359
Online tests or other tools to help gauge if there is an AOD problem^c^	42.6 (266)	624	52.6 (190)	361
A quick and easy user profile system that tailors information to need	42.7 (268)	628	44.2 (159)	360
Downloadable fact sheets for friends^b^	37.4 (232)	620	44.9 (162)	361
Prevention programs for those “at risk” of developing an AOD problem^c^	34.9 (217)	622	48.5 (174)	359
Downloadable fact sheets for family or carers^b^	35.7 (222)	621	45.9 (166)	362
An online treatment program with assistance (phone, IM, email or webcam)	32.0 (199)	622	32.7 (118)	361
A tracking function^b^	26.8 (167)	624	35.9 (129)	359
An online self-help treatment program ^a^	25.7 (160)	622	33.1 (120)	362
A consumer information sharing hub to share experiences	29.5 (184)	624	25.3 (92)	363
Material/text presented in a different language	10.9 (68)	623	12.5 (45)	360
A chat room	9.8 (61)	625	8.9 (32)	361
Being able to start up your own online support group	8.9 (55)	619	9.2 (33)	358

^a^ P < .05

^b^ P < .01

^c^ P < .001

Alcohol website users were significantly more likely to endorse a range of tools/functions as very important in comparison with the drug website users, specifically: online screening tools (χ²_2_ = 15.8, *P* < .001, n = 985); prevention programs (χ²_2_ = 27.5, *P* < .001, n = 981); tracking functions (χ²_2_ = 11.5, *P* = .003, n = 983); self help treatment programs (χ²_2_ = 8.3, *P* = .02, n = 984); downloadable fact sheets for friends (χ²_2_ = 11.6, *P* = .003, n = 981); and family (χ²_2_ = 12.7, *P* = .002, n = 983). In addition, 24 respondents added the following other important tools/functions: the provision of a blend of positive and negative personal testimonials, good information, and peer reviewed journal articles.

Preferences for specific website tools or functions did not vary by gender. However, several significant differences were found for age and higher educational level. Age group impacted on the Web portal feature (χ²_10_ = 29.1, *P* = .001, n = 962) with respondents between 16 and 24 years of age valuing a Web portal least and those between 44 and 61 years valuing it most. Those in the youngest age group also valued access to a chat room most, and those aged 44 to 52 years placed least importance on this feature (χ²_10_ = 30.4, *P* = .001, n = 967). The youngest age group also valued a consumer hub the most, and those aged 33 to 52 years valued it least (χ²_10_ = 34.6, *P* < .001, n = 968). Those in the youngest age category valued the ability to start up an online support group most, and those aged 44 to 52 placed the least importance on this feature (χ²_10_ = 26.97, *P* = .003, n = 958). However, being able to download fact sheets for family and carers was most important to those aged 43 to 52 and least important to those aged 16 to 24 (χ²_10_ = 49.6, *P* < .001, n = 964).

Respondents without higher education valued support groups most, and those completing an undergraduate degree valued support groups least in comparison with other education groups (χ²_16_ = 27.1, *P* = .04, n = 708). Access to therapist-assisted online treatment programs was also influenced by the respondents higher education level (χ²_16_ = 27.9, *P* = .03, n = 713), with those holding a diploma as their highest tertiary educational qualification valuing therapist-assisted online treatment programs most highly and those undertaking a degree valuing them least. Finally, respondents with a diploma, an apprenticeship, or no higher education valued the presentation of website content in another language the most, and those currently completing an undergraduate degree valued this the least (χ²_16_ = 27.7, *P* = .03, n = 714).

### Preferred Support Mode for Alcohol and Drug Problems

Respondents were asked to consider what type of online service they would prefer if they had an alcohol or drug problem. As shown in Table 6, the most highly preferred service mode for either problem was a website with email support from a therapist, and the least preferred was a website with no support or with telephone support from a therapist. No significant differences were found between the alcohol and drug website user groups on preferred support options (Drug problem: χ²_4_ = 5.2, *P* = .26, n = 994; Alcohol problem: χ²_4_ = 1.5, *P* = .82, n = 994).

**Table 6:** Most preferred support mode if the respondent had a drug or alcohol problem (n = 994)

Type of Treatment Support	Treatment for Drug Problem		Treatment for Alcohol Problem	
	Drugs (n = 629) % (n)	Alcohol (n = 365) % (n)	Drugs (n = 629) % (n)	Alcohol (n = 365) % (n)
Website with email support from a therapist	34.0% (214)	33.4% (122)	34.8% (219)	36.2% (132)
Website with face-to-face support from a therapist	23.7% (149)	29.0% (106)	21.0% (132)	21.9% (80)
A self-help website with no therapist support	18.9% (119)	15.3% (56)	20.3% (128)	17.8% (65)
Website with telephone support from a therapist	17.8% (112)	18.1% (66)	18.0% (113)	19.2% (70)
Other (Please specify)	5.6% (35)	4.1% (15)	5.9% (37)	4.9% (18)

Respondents were again able to specify other preferred support options and 55 respondents did so. The most common responses were “all the above,” only wanting to see a healthcare practitioner on a face-to-face basis without a treatment website, an alcoholics/narcotics anonymous online group, synchronous online one-to-one counselling, and an Internet site with chat room support.

No demographic variables were found to impact upon preferences for online treatment and supports.

## Discussion

This study was the first to capture information on the content and functionality preferences of AOD and health-related websites users in Australia. Consistent with previous research on health-related websites, such as the study by Brouwer et al [18], fundamental website features relating to design and navigation were highly valued. The most common reason for using an AOD website was to obtain information about alcohol and other drugs, and approximately half of the respondents found information they wanted in 5 to 15 minutes. It is unclear whether this result was because website writers paid insufficient attention to the provision of information that users want; was due to deficiencies in website design, navigation, and search functions; or reflected problems with the written expression, structure, or layout of the material. However, consistent with an informational focus, open access, the right amount of information, easily understood language, no need for additional software, and an ability to download or print material were accordingly seen as particularly important.

Presentation was also important: approximately half the sample highly valued interesting Web pages, pictures and graphics, and external links, although video, audio, and flash or animations were far less preferred. Being able to ask a question was also valued by more than half of respondents, but other interactive features such as quizzes and online games were highly valued by less than 20% of the sample. This clearly raises important questions for website developers with respect to the prioritization of elements and features to be incorporated, particularly given the high cost of games, videos, and animations.

Respondents were more likely to reply that website trustworthiness was primarily indicated by evidence, cited sources, expertness of its writers, and documented currency, rather than by recommendations or seals of approval. These results suggest that this sample of users was relatively sophisticated in its ability to judge the quality of websites for themselves.

Alcohol website users were more likely than respondents who had accessed sites on other drugs to highly value online screening tools, prevention and self-help treatment programs, tracking functions, and fact sheets for family and friends. It is unclear why this result was found, and in particular, whether it reflected a greater willingness in this sample to consider addressing problems with alcohol than addressing problems with other drugs. Replication of the result and further investigation of its implications for website marketing and design are needed. In other respects, the alcohol and other drug samples provided very similar responses.

Gender did not affect any survey responses. However, there were several differences across age and education groups. Notably, younger people were more likely to value interactive and social networking features (being able to ask a question, the consumer hub, chat room access, ability to set up an online support group, games, and the option to blog). This result may reflect the greater likelihood among younger website users to turn to peers when seeking information or support. In contrast, older respondents valued the site map, the Web portal and the ability to download information or fact sheets for family and friends more highly. The value placed on the ability to access information may reflect a greater likelihood among this group that they were caring for someone with a drug or alcohol problem, and, therefore, valued high-quality information with which to assist these people. It may also reflect a greater reliance among older respondents on expert information about alcohol and other drugs in preference to obtaining this information from peers. Interestingly, respondents without higher education valued having a support group most, and those undertaking a degree valued online treatment least. It is not clear whether less educated respondents were more likely to see their existing support system as inadequate or whether they felt less able to address alcohol or other drug issues on their own. Respondents undertaking a degree also valued multilingual options least (presumably because higher education in Australia is typically conducted in English).

Overall, these demographic differences suggest that website developers should consider the characteristics of the intended target group when designing a website. Greater understanding of each of the results is needed to know how best to address the differences in perceived needs.

Lastly, we were interested in knowing what type of online support was most preferred by the survey respondents if they were to require treatment for either a drug or alcohol problem. An Internet site with email support from a therapist was the most preferred option by both AOD website user groups, although this was selected by less than a third of respondents, with other selections being almost evenly spread across the other options. This survey did not directly compare Internet options with face-to-face treatment alone. However, as very few AOD groups endorsed the “other” option for support and only a subset of these identified standard face-to-face therapy as their preferred type of support, this suggests that standard therapy was not salient and that respondents to an online survey may be willing (and may even prefer) to use online treatment modalities should they require treatment. It is particularly noteworthy that neither the type of website accessed nor respondent demographics impacted upon the preferred type of online support. It seems that an important component of an online “treatment” program could potentially be the provision of some therapist support, especially via email.

### Limitations

Survey respondents were primarily a young, educated, English speaking, and employed Australian sample who used the Internet on a daily basis. Relative to the Australian population at the time of the survey, our sample had more women (66% vs 51%), was younger (median age 22.0 years vs median of 36.9 years), had higher participation rates in employment (75% vs 65.2% of 15-74 year olds), and had higher rates of post school qualifications (7% vs 31% with no tertiary qualifications) [20]. However, the percentage of respondents for whom English was a second language (14%) was identical to the percentage in the Australian general population. 

The interaction between these sociodemographic variables and health seeking/health literacy is well documented. For example, education can increase the likelihood of employment, thereby affecting the means by which people can improve their health and well-being as well as their ability to understand and choose pathways to better health [21]. Higher rates of health literacy are also found among people with higher levels of education and who are employed versus unemployed [21].The representativeness of the sample may have been further compromised by the online nature of the survey (ie, self selecting bias) and where it was marketed (eg, Facebook). The implications of these differences in terms of generalizability may also be similar to those encountered when conducting other Internet-based research. For example, in Australia, Internet access is highest amongst people with higher incomes, higher levels of educational attainment, and in households with children over the age of 15 years, with younger age groups reporting higher levels of Internet usage compared with those aged 55 years and over [22]. Thus, our survey respondents may more closely resemble Australian Internet users than the wider population. Furthermore, our recruitment methods may have attracted a sample of respondents with greater experience and knowledge of AOD websites than the general population of Internet users. These features can be seen as both a limitation (results may not be generalized to the whole Australian population) and a strength (they may more closely match Internet users, especially those who access AOD sites). An additional, related limitation is that the sample was entirely Australian, and results may not be fully generalizable to AOD respondents from other countries.

Respondents answered the questions based on websites they had visited before. There was no control for variability in website exposure (eg, type, frequency, or recency of previous website visits). Nor were motivations for seeking information standardized. So, respondents may have been seeking information about their own substance use, about family members or friends, or may have been seeking information for study programs or for entertainment. Differing features may have been seen as important or satisfactory based on these differing agendas. Perhaps more importantly, we do not know whether any of the respondents had an AOD use problem (either in the past or currently) and accordingly, we do not know how these results may relate to an AOD treatment-seeking population.

### Future Research

Future researchers might consider conducting a comparison of similarly motivated individuals seeking websites on AOD issues. Developing and evaluating AOD websites based upon the specifications identified as highly important by this sample could lead to higher rates of engagement and usage of AOD websites. In addition, exploration of some of the functional design issues, such as determining what amount of information is considered the “right” amount could assist in the design of a more user-friendly AOD website. Targeting people with AOD use problems would also provide more specific information regarding the wants and needs of this group in accessing online AOD websites. Tailoring AOD websites based on age and education level appears to be an important line of research investigation, and whether gender is indeed an irrelevant factor when developing AOD websites will be an important finding to replicate.

### Implications and Conclusion

Engagement of people with AOD problems in behavior change remains a difficult challenge. The Internet offers opportunities both for increased community understanding of AOD issues, and potentially, for engagement of affected individuals at an earlier stage than traditional treatment services, with lower stigma, and at less cost. However, community-wide Internet sites have not engaged young people at the rates one might have wished, with alcohol-related website users often having mean or median ages in the mid 40s, as in the study of Kramer et al, for example [23]. The results of the current study suggest some partial solutions. For example, the results suggest that both younger and older website users may be attracted to sites that offer easily understood information, particularly if it can be easily accessed, as well as sites where there is no need to log in or download additional software and there are opportunities to ask questions. While websites should be attractive and easy to use, high cost features such as games, animations, or videos are not required by the majority. Users may at first be tempted to look at screening tools or tips, although once engaged, we found that users showed a preference for therapist assistance over stand-alone websites (a preference that may limit the ability of the websites to fully realize their potential for reaching the community at low cost). The results of the study also suggests that the challenge of eliciting a transition from information seeking to screening and treatment-seeking may be greater for other drug website users than for alcohol website users, although that apparent effect requires replication and clarification.

## Multimedia Appendix 1

Online alcohol and drug websites: What do you think?

## Multimedia Appendix 2

Alcohol and drug websites

## Abbreviations

AOD: alcohol and other drugs
IP: Internet protocol
SMS: short message service

## References

[1] (2009). International Telecommunication Union.

[2] Jones S, Fox S (2009). Generations online in 2009.

[3] Evers KE (2006). eHealth promotion: the use of the Internet for health promotion. Am J Health Promot.

[4] Lieberman DZ, Huang SW (2008). A technological approach to reaching a hidden population of problem drinkers. Psychiatr Serv.

[5] Linke S, Murray E, Butler C, Wallace P (2007). Internet-based interactive health intervention for the promotion of sensible drinking: patterns of use and potential impact on members of the general public. J Med Internet Res.

[6] Copeland J, Martin G (2004). Web-based interventions for substance use disorders: a qualitative review. J Subst Abuse Treat.

[7] Cunningham JA, Wild TC, Bondy SJ, Lin E (2001). Impact of normative feedback on problem drinkers: a small-area population study. J Stud Alcohol.

[8] Linke S, McCambridge J, Khadjesari Z, Wallace P, Murray E (2008). Development of a psychologically enhanced interactive online intervention for hazardous drinking. Alcohol Alcohol.

[9] Saul JE, Schillo BA, Evered S, Luxenberg MG, Kavanaugh A, Cobb N, An LC (2007). Impact of a statewide Internet-based tobacco cessation intervention. J Med Internet Res.

[10] Cloud RN, Peacock PL (2001). Internet screening and interventions for problem drinking: Results from the http://www.carebetter.com. Alcohol Treat Q.

[11] Cunningham JA, Humphreys K, Koski-Jännes A (2000). Providing personalized assessment feedback for problem drinking on the Internet: a pilot project. J Stud Alcohol.

[12] Linke S, Brown A, Wallace P (2004). Down your drink: a web-based intervention for people with excessive alcohol consumption. Alcohol Alcohol.

[13] Koski-Jännes A, Cunningham J, Tolonen K (2009). Self-assessment of drinking on the Internet--3-, 6- and 12-month follow-ups. Alcohol Alcohol.

[14] Humphreys K, Klaw E (2001). Can targeting nondependent problem drinkers and providing internet-based services expand access to assistance for alcohol problems? A study of the moderation management self-help/mutual aid organization. J Stud Alcohol.

[15] Riper H, Kramer J, Keuken M, Smit F, Schippers G, Cuijpers P (2008). Predicting successful treatment outcome of web-based self-help for problem drinkers: secondary analysis from a randomized controlled trial. J Med Internet Res.

[16] Eysenbach G, Köhler C (2002). How do consumers search for and appraise health information on the world wide web? Qualitative study using focus groups, usability tests, and in-depth interviews. BMJ.

[17] Danaher BG, McKay HG, Seeley JR (2005). The information architecture of behavior change websites. J Med Internet Res.

[18] Brouwer W, Oenema A, Crutzen R, de Nooijer J, de Vries NK, Brug J (2008). An exploration of factors related to dissemination of and exposure to internet-delivered behavior change interventions aimed at adults: a Delphi study approach. J Med Internet Res.

[19] Napolitano MA, Fotheringham M, Tate D, Sciamanna C, Leslie E, Owen N, Bauman A, Marcus B (2003). Evaluation of an internet-based physical activity intervention: a preliminary investigation. Ann Behav Med.

[20] Australian Bureau of Statistics (2009). Australian Bureau of Statistics.

[21] Australian Bureau of Statistics (2009). Australian Bureau of Statistics.

[22] Australian Bureau of Statistics (2009). Australian Bureau of Statistics.

[23] Kramer J, Riper H, Lemmers L, Conijn B, van Straten A, Smit F (2009). Television-supported self-help for problem drinkers: a randomized pragmatic trial. Addict Behav.

