# Supplementary material for: Content and Functionality of Alcohol and Other Drug Websites: Results of an Online Survey
Source: J Med Internet Res. 2010 Dec 19;12(5):e51. doi: 10.2196/jmir.1449 (PMC3057306; doi:10.2196/jmir.1449)
Supplement: Supplementary file 1 [file jmir_v12i5e51_app1.pdf]

Multimedia Appendix 1. Online alcohol and drug websites: What do you think?

Online alcohol and drug websites – What do you think?

What is it about?
The Department of Health and Ageing (DoHA) has contracted QUT to investigate the existence and usage of internet sites providing information about alcohol and other drugs and co morbidity to Australians.

As part of this research we are conducting an online survey investigating people views and opinions about alcohol and drug websites. In particular, we are interested in what people like and don't like about these kinds of health-related information or treatment websites.
The survey will take approximately 10 minutes.
Everyone who completes the survey can enter the draw for an 8GB IPOD Nano.

Can I do the survey?
If you are 16 years or older we would like to invite you to participate in the survey.
You do not have to have had any personal experience with alcohol or drug websites to participate in the survey.

What do I need to do?
If you are interested in participating in this survey, please follow the link to the survey.

http://tinyurl.com/alcoholanddrugsurvey
